# Spectrophotometric resolution for quantitative analysis of aspirin and rivaroxaban combination therapy in biological fluids using simple and eco-friendly procedure

**DOI:** 10.1186/s13065-024-01140-3

**Published:** 2024-02-19

**Authors:** Heba M. Mohamed, Hebatallah M. Essam

**Affiliations:** Analytical Chemistry Department, Faculty of Pharmacy, Cairo University, Kasr Al-Aini St., Cairo, 11562 Egypt

**Keywords:** Aspirin, Rivaroxaban, Peripheral artery disease (PAD), Biological fluid, Greenness assessment

## Abstract

Patients diagnosed with symptomatic peripheral artery disease (PAD) in the lower extremities have a higher likelihood of suffering from major vascular events. Recently, FDA has approved the combination therapy of aspirin (ASP) and rivaroxaban (ROX) to reduce acute limb ischemia and other comorbidities in (PAD) patients. Zero order and ratio absorption spectra were employed in three simple and accurate spectrophotometric techniques (dual wavelength (DW), ratio difference (RD) and derivative ratio (^1^DD) for concurrent detection and quantification of ASP and ROX in their pure forms, lab synthetic mixtures and in biological fluid. Our approach involves careful parameter optimization, including solvent selection, sample volumes, and instrumental settings, to reduce the analysis environmental impact. The acquired recovery percentages of accuracy were within 98–102% for pure active pharmaceutical ingredients and 90–110% for pharmaceutical formulations and biological determinations. A comprehensive assessment was done to compare the three methods regarding their ease of use, linearity, sensitivity, conditions, and limitations. The specificity of the proposed methods was evaluated by analyzing the lab synthetic mixtures. The suggested spectrophotometric methods were validated in compliance with ICH guidelines to confirm the validity claims. Also, statistical analysis was done to compare the outcomes obtained from the suggested methods with those obtained from the official ones and they agreed with null hypothesis regarding accuracy and precision. Furthermore, a comprehensive assessment of the environmental sustainability of the developed method was carried out using the Analytical Greenness Calculator, AGREE algorithm. The selected drugs can be efficiently, safely and economically analyzed by the suggested methods in pharmaceutical and biological matrices with no pretreatment or preliminary separation steps and thereby increasing their greenness level.

## Introduction

**Fig. 1:**
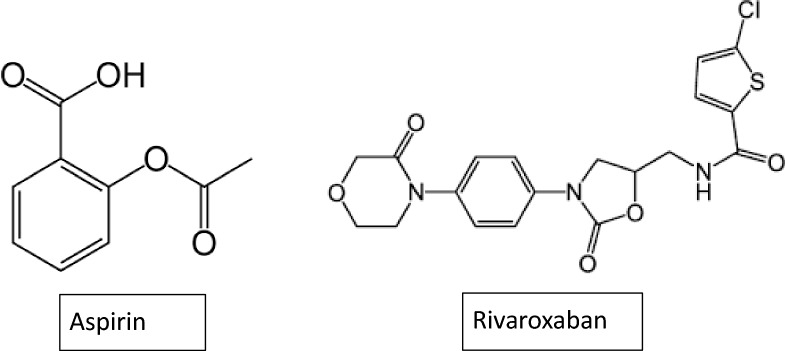
Chemical structure of Aspirin and Rivaroxaban

Peripheral artery disease (PAD) is a serious condition that often goes disregarded or even unaddressed by both patients and healthcare providers due to insufficient awareness and other health issues that take the precedency [1]. Globally, about 200 million individuals are affected by peripheral artery disease (PAD), which is related to the considerable cardiovascular adverse events, morbidity, and mortality [2, 3]. If PAD is timely diagnosed and managed, cardiovascular risks can be highly decreased and maintained [1]. Recently in 2023, Janssen Pharmaceuticals (Johnson & Johnson) has revealed data from phase 3 VOYAGER PAD clinical trial, which supports the benefits of prescribing rivaroxaban in combination with aspirin in the management of disease over the use of aspirin alone (the current standard care) [4]. This combination therapy becomes the first approved one by FDA in April 2021 [5, 6]. In addition, this combination provides 33% reduction in acute limb ischemia following Lower Extremity Revascularization (LER) [5]. Aspirin, [2-(acetyloxy) benzoic acid] or acetylsalicylic acid, is one of the non-steroidal anti-inflammatory drugs (NSAID) that exhibits anti-inflammatory, analgesic, and antipyretic effects. Aspirin, structure shown in Fig. 1, is also widely used for its antithrombotic properties, which result from its ability to reduce platelet aggregation [7]. Rivaroxaban, structure shown in Fig. 1, is an oral anticoagulant, a potent, selective, and direct inhibitor of factor Xa that prevents thromboembolism during surgical operations [8]. Rivaroxaban has demonstrated efficacy in both preventing and treating venous thromboembolism, also, it can be used to prevent stroke in individuals with atrial fibrillation. Furthermore, rivaroxaban is the only anticoagulant approved so far to be taken in combination with aspirin to reduce the risk of major thrombotic vascular events in peripheral artery disease (PAD) patients. It is also worth mentioning that in some cases fatal adverse effects related to rivaroxaban combined with aspirin were reported in some patients which recommends the proper monitoring of coagulation during rivaroxaban treatment along with proper attention to dosage error [9, 10].

Aspirin had been analyzed either in single formulation or in multi-component medications using UV–VIS spectrophotometry [11–16], chromatography; HPLC [17–24], UPLC [25] and TLC methods [26, 27] and electrochemical analysis [28, 29]. While, for rivaroxaban, the literature shows determination of ROX by spectrophotometric methods [30, 31] and many chromatographic methods for quantification of ROX in its dosage form via HPTLC [32, 33], HPLC [34–36] and in plasma [37, 38]. Electrochemical behavior investigation using square-wave voltametric to determine rivaroxaban in pharmaceutical dosage forms was also reported [39].

Since this combination therapy was just approved in March 2023, literature check showed no publications of simple and accurate spectrophotometric methods to analyze this novel therapeutic protocol. Therefore, the aims of this work are, first to develop and validate three smart and simple spectrophotometric methods to resolve sever overlapping spectra of ASP and ROX without previous separation. These methods rely on either using the zero-order spectra (dual wavelength) or the ratio spectra (ratio difference and derivative ratio) to resolve the overlap and allow the determination of aspirin and rivaroxaban in their pure forms and laboratory synthetic mixtures containing their therapeutic regime ratio. Furthermore, the suggested Spectro-photometric methods were successfully applied to determine the drugs in biological fluids (human plasma). The methods are simple, accurate and precise, moreover, they do not require any sophisticated apparatus or complicated software. Besides, minimum use of solvents, energy, and hazardous wastes during the whole analysis identifies the suggested methods as economic and eco- friendly ones.

## Experimental conditions

### Apparatus and software

Double beam spectrophotometer: Shimadzu (UV-1900i, Japan) using matched 1.00 cm quartz cells was employed to carry out spectrophotometric measurements. Scans were carried out in the range from 200 to 350 nm at 0.2 nm intervals. Spectra were automatically obtained by Shimadzu UV-Probe 2.62 system software. Centrifuge (Model: 2-16P, Sigma Laborzentrifugen, Germany), PVDF filter (Millex1-GV, Merck Millipore, Cork, Ireland).

### Samples

#### Standards

Aspirin (ASP) was purchased from Sigma laboratories, its purity was tested and found to be 100.22 ± 0.947% as determined by the official titrimetric USP method [40]. Rivaroxaban (ROX) was kindly supplied from Megafine Pharma (P) Ltd., its purity was tested and found to be 100.48 ± 0.915% as determined by reported method [34].

#### Pharmaceutical formulation

Aspirin protect 100 mg® by Bayer Schering Pharma, Germany batch # BTAB235, labeled to contain 100 mg ASP/tablet, was purchased from market.

Xarelto® by Bayer Schering Pharma, Germany batch # NDC 50458–580-30 claimed to contain 10 mg ROX/tablet, was purchased from market.

### Materials and solvents

Methanol and DMSO of HPLC grade, was obtained from Sigma- Aldrich, Darmstadt-Germany.

Blank human plasma samples were obtained from El-Kasr El-aini Hospital Blood Bank and kept frozen until use after gentle thawing.

### Standard solutions

Stock standard solution 100 μg/mL of aspirin in methanol and stock standard solution 100 μg/mL rivaroxaban in methanol: DMSO mixture (95:5) were freshly prepared and stored in the fridge during analysis time.

### Laboratory synthetic mixtures containing different ratios of ASP and ROX

Into a series of 25-mL volumetric flasks, variable aliquots of ASP and ROX were transferred from their corresponding stock solutions (100 μg/mL). Then volumes were completed with methanol to prepare mixtures containing different ratios of the two drugs, including the recommended combination therapy ratio.

## Procedure

### Spectral characteristics

**Fig. 2:**
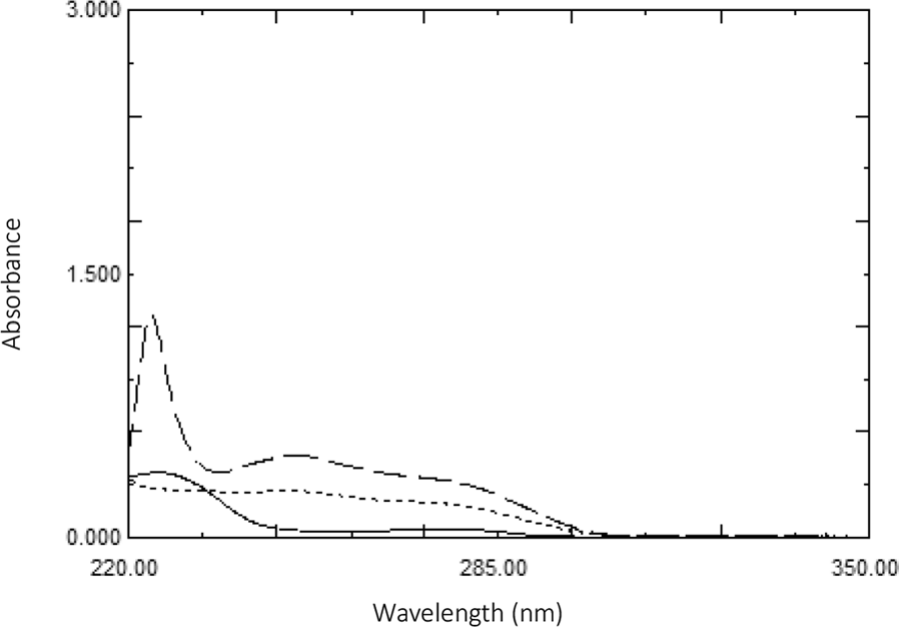
UV zero order absorption spectra of (8 µg/mL) ASP (—) and ROX (- - -) and a mixture of 4 µg/mL each (…..), all in methanol

Zero order absorption spectra of ASP and ROX each of concentration 8 μg/mL and a mixture of both components (4 μg/mL each) in methanol were recorded over the range 200–350 nm, as shown in Fig. 2.

### Spectrophotometric measurements

Aliquots of 1.0, 2.0, 10.0 mL from ASP stock standard solution (100 μg/mL) in methanol and aliquots of 0.5, 1.0, 2.0, 5.0 mL from ROX stock standard solution (100 μg/mL) in methanol: DMSO mixture (95:5) were accurately transferred into a series of 25-mL volumetric flasks and the final volumes were completed with methanol. Then the absorption spectra of these standard solutions were scanned in the specified wavelength range (200–350 nm) using methanol as a blank and saved in computer to be used for subsequent data manipulation and spectral resolution of the two drugs.

#### Spectrophotometric methods using zero-order spectra.

##### Dual wavelength METHOD (DW)

Absorbance differences of zero order absorption spectra (^0^D) of the prepared ASP solutions (4.0–40.0 μg/mL) were measured between 242.5 nm and 255.5 nm, where they show significant values, while those of ROX are zeros. On the other hand, for determination of ROX; the absorbance differences of (^0^D) of its prepared solutions (2.0–20.0 μg/mL) were measured between 252 and 286 nm that correspond to ROX concentrations, while those of ASP are having zero values between the selected wavelengths. Formerly the absorbance difference between the two designated wavelengths for each drug was plotted versus the different ASP and ROX concentration values and the regression equations were computed.

#### Spectrophotometric methods manipulating ratio spectra

##### Ratio difference method (RD)

The stored spectra of ASP and ROX were used to obtain the ratio spectra. For ASP, its absorption spectra were divided by the 20 µg/mL ROX spectrum, while for ROX, its spectra were divided by 36 µg/mL ASP spectrum. The difference between amplitudes of the ratio spectra at the selected wavelength pairs (234 nm & 280 nm for ASP and 254 & 297 nm for ROX) were plotted against the corresponding concentrations of ASP and ROX, respectively to construct the calibration graphs from which the regression equations were computed.

##### Derivative ratio method (^1^DD)

The obtained ratio spectra of ASP & ROX by dividing their zero order spectra by 20 µg/mL ROX spectrum or 36 µg/mL ASP spectrum, respectively, were transformed into the corresponding first order derivative spectra. Derivatization was applied with Δ λ = 4 nm that allow minimum noise with more detailed spectral features, and a scaling factor = 10 to achieve suitable measurements. Peak amplitudes at 238.5 nm and 293 nm were measured and plotted against ASP and ROX concentrations respectively, to construct the calibration graphs from which the regression data was obtained for each drug.

### Analysis of laboratory synthetic mixtures

#### Spectrophotometric methods using zero-order spectra

To determine ASP and ROX by DW method, scan the prepared mixtures in the range of 200–350 nm, measure the differences in absorbance between the selected pair of each drug (242.5 nm & 255.5 nm for ASP and 252 nm and 286 nm for ROX). Then the drugs concentrations were calculated using the corresponding regression equation for each one.

#### Spectrophotometric methods manipulating ratio spectra

For RD method, to determine ASP and ROX, carry on the aforementioned procedures to obtain the ratio spectra of the prepared mixtures, then measure the differences in peak amplitudes between selected wavelength pairs (234 nm & 280 nm for ASP and 254 & 297 nm for ROX). The drugs concentrations were calculated using the corresponding regression equation for each one.

While for ^1^DD method, to determine ASP and ROX, apply the first derivative steps on the obtained ratio spectra for each mixture using the specified condition. Record measurements at 238.5 nm and 293 nm and calculate the drugs concentrations using the corresponding regression equation for each one, respectively.

### Analysis of dosage forms

#### For ASP tablets

Ten Aspirin protect® 100 mg tablets were accurately weighted and finely crushed and homogenously mixed. An amount of powder equivalent to 100 mg of ASP was accurately weighted, transferred into a beaker, 50 mL of methanol was added, then a continuous stirring was applied for 10 min using a magnetic stirrer. The solution was sonicated for 10 min then filtered and accurately transferred into a 100 mL volumetric flask. The volume was completed to the mark by methanol. Additional dilution was carried out to prepare a working standard solution (100 μg/mL) that could be used for subsequent preparations.

Different concentration levels within the linearity range were prepared and analyzed using the described procedures of the suggested methods (DW, RD and ^1^DD).

#### For ROX tablets

Ten Xarelto® 10 mg tablets were accurately weighted and finely crushed and homogenously mixed. An amount of powder equivalent to 10 mg of ROX was accurately weighted, transferred into a beaker, 50 mL of methanol: DMSO mixture (95:5, v/v) was added, and using a magnetic stirrer the beaker was continuously stirred for 10 min. The solution was sonicated for 10 min then filtered and accurately transferred into a 100 mL volumetric flask, then the volume was completed to the mark by the same solvent.

Different concentration levels were prepared along the linearity range and analyzed using the described procedures of the suggested methods (DW, RD and ^1^DD).

For DW, the concentrations of ASP and ROX were calculated by direct substitution of the absorbance difference in their corresponding regression equations. On the other hand, differences in peak amplitude were used for calculations in RD method. Finally, peak amplitudes at the selected wavelengths were substituted in the corresponding regression equations of ^1^DD method.

### Application to biological fluid (human plasma)

As the two drugs ASP and ROX are co-administered together in a specific treatment regime, we aimed to develop a sensitive and simple method for their simultaneous determination in human plasma.

A volume of 500.0 μL plasma was transferred into two screw capped centrifuge tubes and then separately spiked with 4 mL of ASP and ROX laboratory synthetic mixtures (Intermediate mixtures concentrations: 25: 12.5 µg/mL of ASP: ROX and 75: 37.5 µg/mL of ASP: ROX, respectively). 0.5 mL of methanol as a precipitating agent was added to ensure samples full deproteination. Each sample was vortex mixed for 3 min and centrifuged at 4000 rpm for 20 min. The supernatants were filtered through a 0.22 μm PVDF filter. Two mL of the filtrate was transferred into a 10-mL volumetric flask and the volume was completed to mark with methanol to obtain mixtures with final concentration of 4: 2 µg/mL ASP: ROX and 12:6 µg/mL ASP: ROX, respectively. Blank plasma sample was carried out simultaneously, in an identical manner except for the addition of ASP and ROX mixtures. All the spiked plasma samples were analyzed using the aforementioned procedures. The respective regression equations were then used to determine the drugs concentrations.

## Results and discussion

Spectrophotometric methods have proven their superiority and efficiency for quantitative analytical purposes for bi-and/or multi-component mixtures [11, 41–43]. Compared to other analytical procedure such as chromatographic methods; spectrophotometric analysis, can provide easy to apply, accurate, time and cost-effective quantitative analysis procedures for mixtures avoiding chemical pretreatment or sophisticated apparatus criteria, high energy demand, high solvent consumption and influential amount of released hazardous waste [42, 43]. Moreover, spectrophotometric methods can be used to determine the drugs in plasma either directly or after augmentation techniques to reach the maximum blood concentration of a specific analyte [44]. These blessings expand the spectrum of spectrophotometric methods to be used in pharmacokinetics studies and to determine the optimal dose and dosing schedule of a drugs that help achieving the desired therapeutic effect while minimizing the adverse effects.

Variable categorizations can be considered for spectrophotometric methods according to their aims, manipulations, spectral features. Commonly, different spectrophotometric manipulations can be applied for either zero order, normalized or ratio spectra. However, according to multicomponent determinations, mathematical spectrophotometric methods can be classified into five types; signal features enhancement e.g. Derivative; hidden information revealing e.g. Chemometrics; standard addition technique; spiking technique or spectrum addition [41].

To determine ASP and ROX in their novel combination therapy, and to simply address the posed challenge of the significant spectral overlap between ASP and ROX that does not show any chance for clear measurements of neither ASP nor ROX, Fig. 2, it was worthy to find out straightforward approaches that can yield accurate and precise results. Consequently, this work aims to resolve the sever overlapped spectra of ASP and ROX depending on the smart manipulation of the obtained spectra to determine the selected drugs, simultaneously, in their pure forms, laboratory synthetic mixture and in human plasma samples within their dosage regime ratio. This was achieved by applying three handy, precise, and specific methods with minimum data manipulation and sample preparations. Moreover, statistical studies for the ability of the applied methods in resolving complex spectra was constructed to prove the competency of the suggested method with the official and reported ones and to explore any potential source of invalidity.

The binary mixture was resolved using either zero-order spectra by applying the dual wavelength (DW) method or ratio spectra by applying ratio difference (RD) and derivative ratio (^1^DD) methods.

### Spectrophotometric methods using zero-order spectra

#### Dual wavelength method (DW)

The DW method is known for its superb features of being uncomplicated, quick, and precise. It can simply determine the concentration of a particular component in a mixture of other unwanted interfering components. Moreover, the DW method requires minimal data management and consequently, it has a broader range of applications. To utilize this method, the most important requisite is to select a wavelength pair for each drug such that the difference in absorbance is negligible for the interferent and linearly correlated with the target analyte concentrations.

**Fig. 3:**
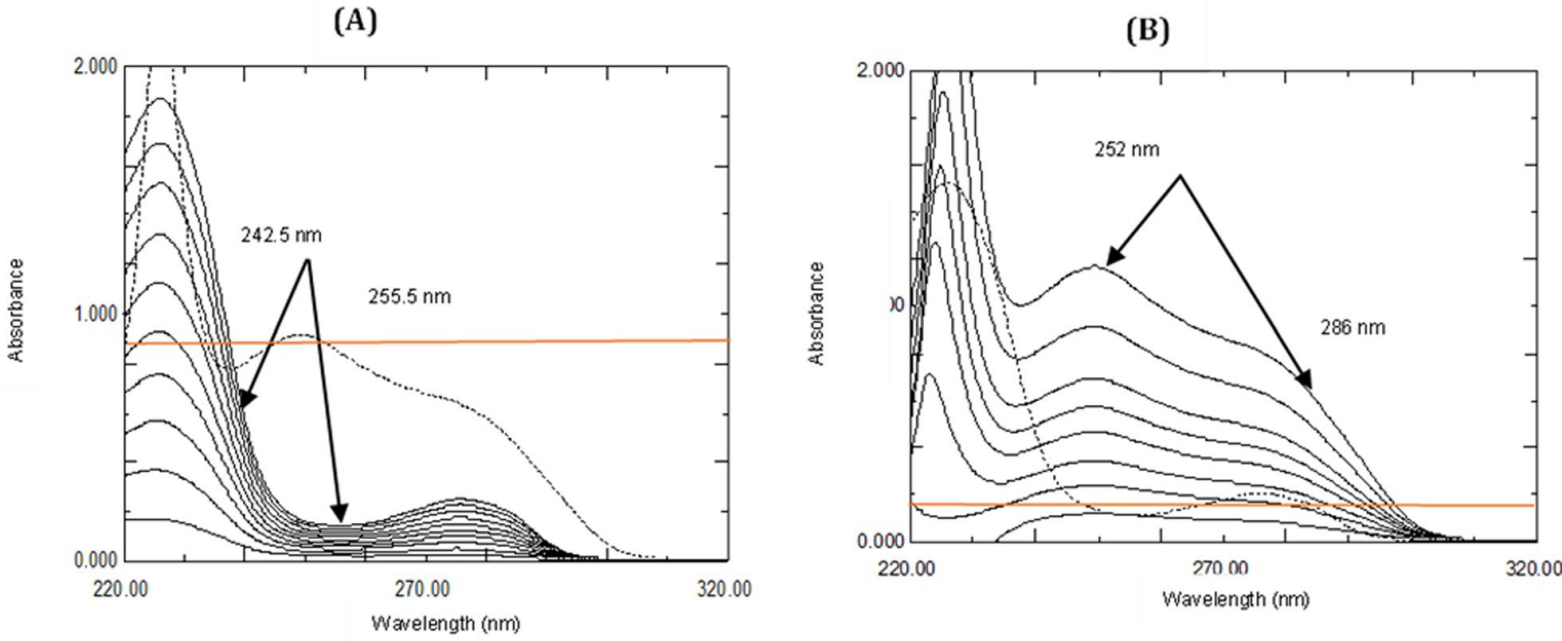
**A** Zero order absorption spectra of 4–40 µg/mL ASP (—) and 16 µg/mL ROX (….). **B** Zero order absorption spectra of 2–20 µg/mL ROX (—) and 32 ug/mL ASP (….)

From the overlapping spectra of ASP and ROX, two specific wavelengths were selected for each drug, as shown in Fig. 3. The absorbance difference at 242.5 nm (λ1) and 255.5 nm (λ2) was found to be zero for ROX so, its contribution can be repealed and the measurements at these wavelengths are directly proportional to the concentrations of ASP, Fig. 3A. Likewise, the absorbance differences at 252 nm (λ3) and 286 nm (λ4) were selected for determination of ROX, where the difference in absorbance at this specific wavelength pair (A_286_ –A_252_) is negligible for ASP, Fig. 3B. The respective regression equation of each drug was obtained by plotting the absorbance difference between either (λ1, λ2) or (λ3, λ4) against the corresponding concentrations of ASP and ROX, respectively. The calculated regression equations were found to be:

$$\Delta {\text{A}}_{{\left( {{255}.{5},{ 242}.{5}} \right)}} = \, 0.00{\text{54C }} + \, 0.00{1}\quad \quad {\text{r}} = 0.{9998}$$

where ΔA _(255.5, 242.5)_ is the absorbance difference of ASP at 255.5 nm and 242.5 nm, C is the concentration of ASP in µg/mL and r is the correlation coefficient.

$$\Delta {\text{A}}_{{\left( {{286},{ 252}} \right)}} = \, 0.0{\text{291C }} + \, 0.000{3}\quad \quad {\text{r}} = 0.{9997}$$

where ΔA _(286, 252)_ is the absorbance difference of ROX at 286 nm and 252 nm, C is the concentration of ROX in µg/mL and r is the correlation coefficient.

The primary downside of DW is its restriction in selecting the wavelength pair for each component that must be limited to those with a constant absorbance of the interfering substance. This necessitates critical measurements; accordingly, any deviation could lead to low sensitivity and accuracy with bad precision. To avoid poor robustness of the method, this drawback has been tackled by applying the ratio difference method that allows constant contribution of the interferent along the whole spectrum.

### Spectrophotometric methods manipulating ratio spectra.

#### Ratio difference method (RD)

In this simple and precise method, the difference between two wavelengths in the ratio spectrum will dispose any interference caused by the component that is used as a divisor [41]. On contrary, RD method allows the determination of a component in its binary mixture at any two wavelengths all over its liner range in the ratio spectrum after using a divisor of the interfering component unlike the DW method that necessitate a constant absorbance of the interfering component at the selected wavelengths.

Upon choosing the divisors, it is crucial to strike a balance between maximum sensitivity and minimal noise. For the analysis of ASP and ROX concentrations in their mixtures, the use of ASP spectrum (36 μg/mL) and ROX spectrum (20 μg/m) as a divisor, yielded the most optimum results in terms of average recovery and RSD percentage.

**Fig. 4:**
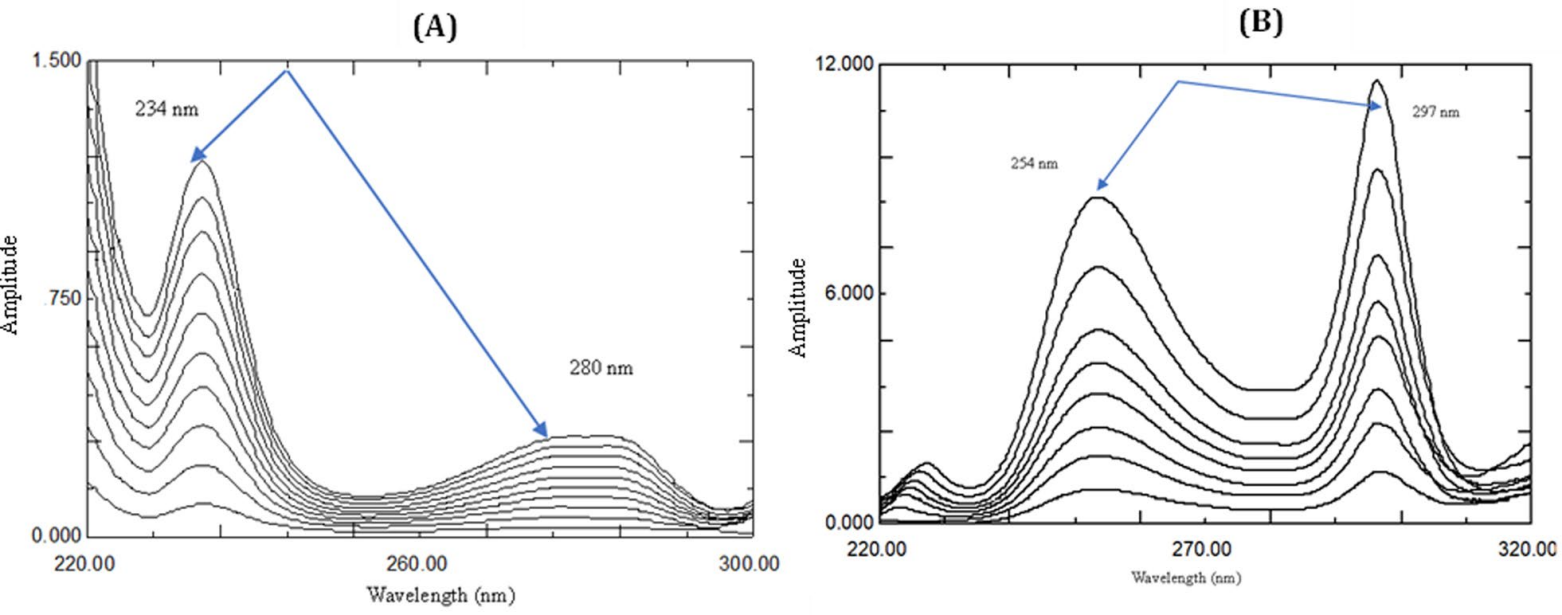
**A** Ratio spectra of ASP 4–40 μg/mL using 20 μg/mL ROX as a divisor in methanol. **B** Ratio spectra of ROX 2–20 μg/mL using 36 μg/mL ASP as a divisor in methanol

To determine ASP and ROX in their binary mixture, firstly, scan the zero order absorption spectra of the mixtures. For ASP determination, thoughtfully a concentration of ROX spectrum (20 µg/mL) was the divisor of choice for the previously scanned spectra. The ratio spectra were produced as shown in Fig. 4A, the amplitudes at 234 nm and 280 nm were measured and subtracted, so the constant contribution of ROX would be cancelled. Then the regression equation used for subsequent determinations of ASP was obtained by plotting the difference in the amplitudes at 234 and 280 nm of the ratio spectra against the corresponding concentrations. The regression equation for ASP was computed and found to be:

$$\Delta {\text{P}} = \, 0.0{\text{221C}} - 0.00{99}\quad \quad {\text{r}} = 0.{9998}$$

where Δ P is the amplitude difference at the two selected wavelengths, C is the ASP corresponding concentration in μg/mL and r is the correlation coefficient.

Similarly, the ratio spectra for ROX determination were produced using ASP spectrum (36 µg/mL) as a divisor and the measurements were recorded at the selected wavelength pair (254 and 297 nm) as shown in Fig. 4B. The regression equation was computed and found to be:

$$\Delta {\text{P}} = 0.{\text{1444C}} + 0.{1495} {\text{r}} = 0.{9999}$$

where Δ P is the amplitude difference at the two selected wavelengths, C is the ROX corresponding concentration in μg/mL and r is the correlation coefficient.

#### *Derivative ratio method (*.^*1*^*DD)*

The derivative ratio spectra method relies on the step of transformation of the ratio spectrum into its first derivative to obtain a spectrum that is independent of the concentration of the interferent. Then measure the amplitude signals of the derivative ratios of the target analyte without any intervention from the other component in the mixture.

**Fig. 5:**
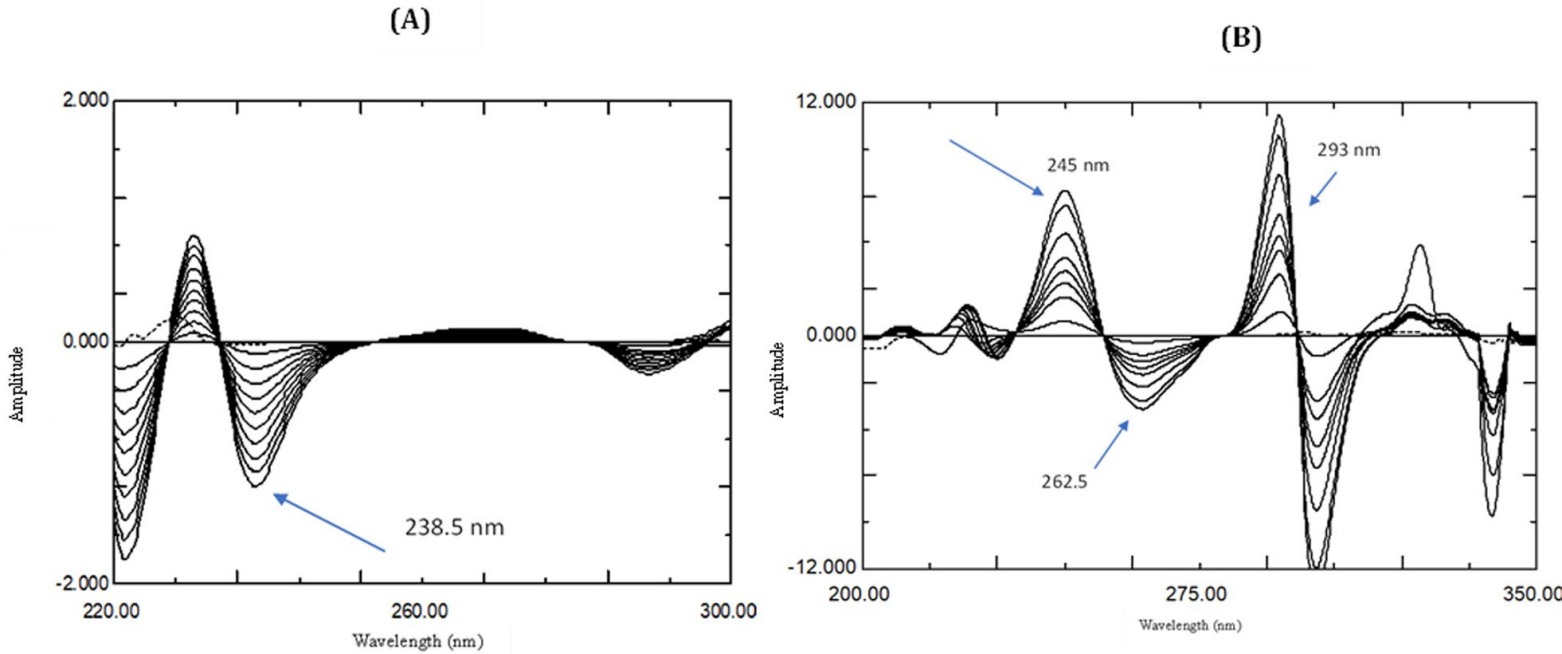
**A** ^1^DD amplitudes of 4–40 µg/mL ASP (—) using 20 µg/mL ROX (….) as a divisor. **B** ^1^DD amplitudes of 2–20 µg/mL ROX (—) using 36 µg/mL ASP (….) as a divisor

For ASP determination, the first derivative of ratio spectra of different concentrations of ASP that obtained using ROX absorption spectrum (20 µg/mL) as a divisor were scanned, then the amplitudes at 238.5 nm were measured that correspond to linear correlation with ASP concentrations as shown in Fig. 5A.

The calibration graph relating the peak amplitudes values at 238.5 nm to the corresponding ASP concentrations was constructed and regression equation was computed and found to be:

$${\text{P}}_{{{238}.{5}}} = - 0.0{3}0{\text{6C}} + 0.0{277}\quad \quad {\text{r}} = 0.{9999}$$

where P _238.5_ is the ASP peak amplitudes at 238.5 nm, C is the concentration of ASP in µg/mL and r is the correlation coefficient.

Similarly, for ROX determination, the first derivative of the ratio spectra, obtained using ASP septum (36 µg/mL) as a divisor, were scanned. Several amplitude peaks and troughs of ROX were observed at 245, 262.5 and 293 nm that allow direct determination of ROX without any interference from ASP. All showed good correlation (r > 0.999), yet 293 nm was the wavelength of choice considering the fact that it showed the highest sensitivity, as in Fig. 5B.

The calibration graph relating the peak amplitudes values at 293 nm to the corresponding ROX concentrations was constructed and regression equation was computed and found to be:

$${\text{P}}_{{{293}}} = 0.{5}0{\text{56C}} + 0.{1667}\quad \quad {\text{r }} = \, 0.{9999}$$

where P _293_ is the ROX peak amplitudes at 293 nm, C is the concentration of ROX in µg/mL and r is the correlation coefficient.

For the laboratory synthetic mixtures analysis, zero-order spectra were divided by ROX (20 µg/mL) followed by first derivative, and the peak amplitudes measured at 238.5 nm were used to calculate ASP concentrations using the specified regression equation. Then similarly, zero-order spectra were divided by ASP (36 µg/mL) spectrum followed by first derivative and peak amplitudes measured at 293 nm were used to calculate ROX concentration using the specified regression equation.

The regression parameters (slope, intercept, and correlation coefficient) were examined to detect the impact of the divisor concentration. Different divisors were tried and ASP (36 µg/mL) and ROX (20 µg/mL) were found the optimum ones with regard to baseline noise and measurements sensitivity.

The main concept of the chosen spectrophotometric methods is to quantify the target analyte within the studied matrices simply and efficiently without releasing more hazardous chemicals into our biosphere. The most remarkable advantage of DW method is it relies on the intrinsic features of the zero-order spectrum of the analyte. This value makes the determination more reliable, simple and accurate. However, the RD method requires one more graphical manipulation step to obtain the ratio spectra, but it allows more choices for wavelength pairs for the determinations since the interference contribution remains constant. Furthermore, 1DD method has a superior merit over the derivative procedure in which it does not need zero crossing or coincidence point for quantitation as well as it creates new sensitive analytical signals (peak or trough) that increase results sensitivity [45]. Additionally, the availability of many maxima and minima provides an extra benefit in detecting the analyte of interest in the presence of other interfering compounds and/or excipients during the assay.

## Method validation and statistical analysis

The ICH guidelines for method validation [46] were followed with explicit assumptions of accuracy and precision based on the obtained data. The demonstrated results in Table 1 support the claims of validity of the proposed spectrophotometric methods.

**Table 1:** Analytical performance data for ASP and ROX determination using the developed spectrophotometric methods

Parameter	ASP			ROX		
	DW	RD	1DD	DW	RD	1DD
λ (nm)	242.5 & 255.5	234 & 280	238.5	252 & 286	254 & 297	293
Range (µg/mL)	4-40 µg/mL			2–20 µg/mL		
Linearity						
Slope	0.0054	0.0221	− 0.0306	0.0291	0.1444	0.5056
Intercept	0.001	− 0.0099	0.0277	0.0003	0.1495	0.1667
Correlation coefficient	0.9997	0.9997	0.9999	0.9995	0.9998	0.9998
Accuracy^a^						
Mean ± RSD	100.26 ± 1.057	100.72 ± 0.957	99.30 ± 0.799	99.91 ± 0.621	101.38 ± 0.490	99.57 ± 0.614
Precision (% RSD)						
Repeatability^b^	1.132	1.078	1.213	1.037	1.096	1.158
Intermediate precision ^c^	1.288	1.319	1.499	1.395	1.214	1.361
Limits						
Limit of detection, LOD	0.61	0.66	0.53	0.45	0.29	0.26
Limit of quantitation, LOQ	1.85	2	1.63	1.37	0.92	0.79

^a^Accuracy was checked using concentrations (10, 14, 22 μg/mL) for ASP and (5, 9, 11 μg/mL) for ROX

^b^and ^c^Are the intraday and inter-day respectively (n = 3) relative standard deviation of concentrations (4, 16, 24 μg/mL) for ASP and (4, 8, 12 μg/mL) for ROX

### Analytical application of the proposed spectrophotometric methods for dosage forms

The proposed methods were successfully implemented to determine ASP and ROX in their commercial tablets. Application of standard addition technique was done to confirm the accuracy of the three methods as stated in Table 2.

**Table 2:** Determination of ASP and ROX in laboratory-prepared mixtures, marketable sample, human plasma and application of standard addition technique

Sample	Method		
	DW	RD	1DD
ASP			
Laboratory prepared mixtures (n = 5)*	100.12 ± 0.871	99.93 ± 0.854	99.77 ± 1.221
Aspirin protect 100 (BTAB235)	99.21 ± 0.699	99.79 ± 1.042	99.98 ± 1.525
Standard addition of dosage form	98.08 ± 0.943	99.73 ± 1.644	100.82 ± 0.650
Human Plasma^a,^**			
4 µg/mL	99.49 ± 0.805	100.87 ± 0.515	99.81 ± 1.214
12 µg/mL	99.16 ± 0.953	98.85 ± 1.146	100.44 ± 0.589
Standard addition of plasma^a,^**	101. 01 ± 0.484	100.04 ± 1.111	99.42 ± 0.921
ROX			
Laboratory prepared mixtures (n = 5)*	99.88 ± 1.077	99.93 ± 0.854	99.77 ± 1.221
Xarelto 10 mg (NDC 50458-580-30)	100.74 ± 0.952	100.50 ± 1.013	100.80 ± 0.437
Standard addition of dosage form	100.67 ± 1.363	99.95 ± 1.040	98.96 ± 0.491
Human Plasma^a^**			
2 µg/mL	100.24 ± 1.002	99.59 ± 0.892	100.38 ± 0.911
4 µg/mL	100.05 ± 1.071	100.91 ± 0.819	101.06 ± 0.794
Standard addition for plasma^a,^**	99.92 ± 1.447	100.12 ± 1.589	100.30 ± 1.033

^*^5 sets for each method, each of 3 replicates

^**^Mean ± SD %

^a^Each result is the average of three separate determinations

### Analytical application of the proposed spectrophotometric methods to spiked human plasma

The proposed methods were effectively applied for the determination of the novel combination therapy of ASP and ROX in human plasma. Plasma samples typically require deproteinization to eliminate matrix effect. We used a simple liquid/liquid extraction step which was very suitable for our suggested methods as the standards, and the binary mixtures are dissolved in the organic solvent that will help the deprotonation of the plasma without any additional reagents or procedures.

**Fig. 6:**
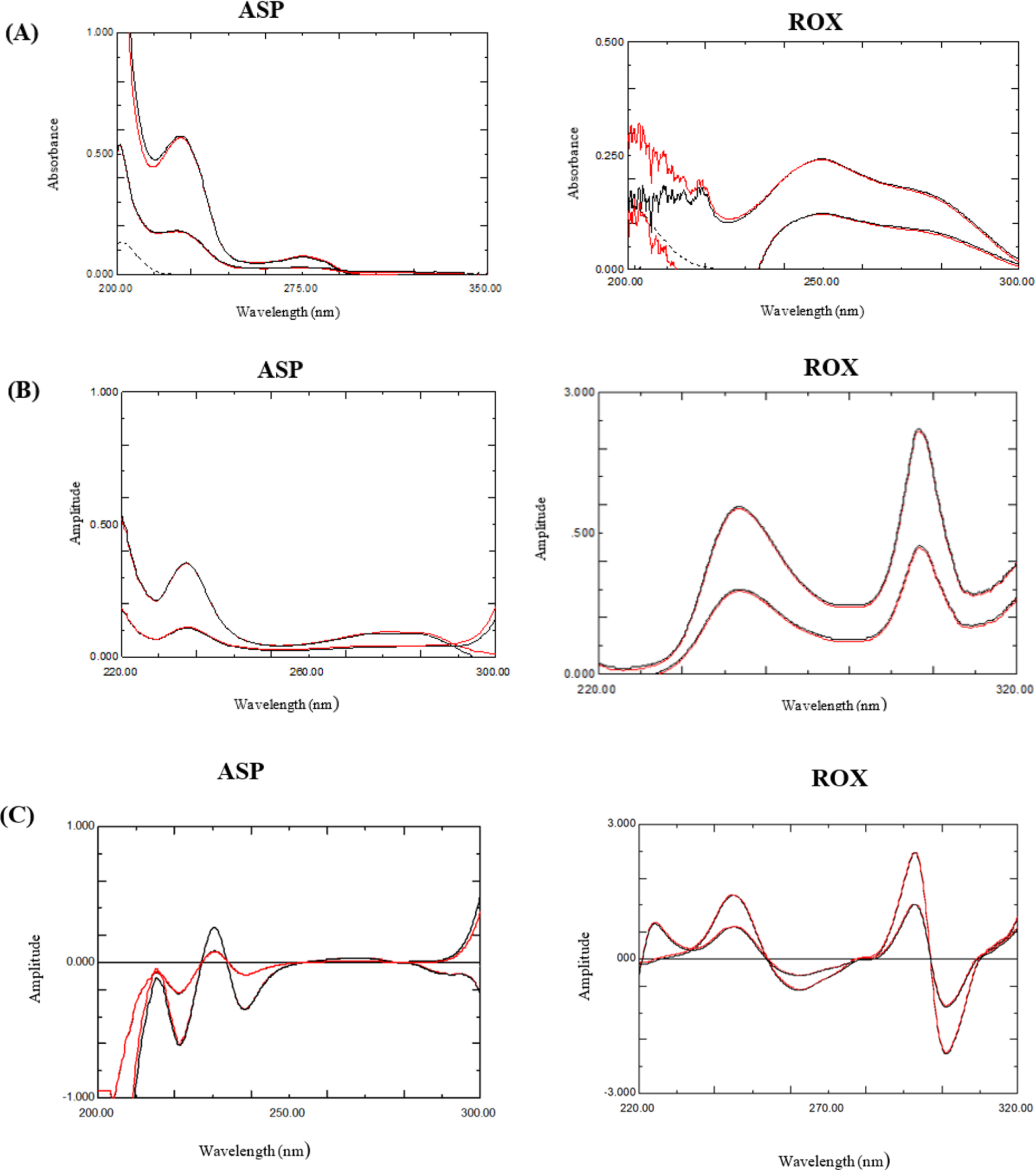
**A** Zero order absorption spectra of Blank plasma (….), 4 and 12 µg/mL ASP in methanol (black) and in plasma (red). And 2 and 4 µg/mL ROX in methanol (black) and in plasma (red). **B** Ratio spectra of 4 and 12 µg/mL ASP in methanol (black) and plasma (red) using ROX 20 µg/mL as divisor. And 2 and 4 µg/mL ROX in methanol (black) and plasma (red) using ASP 36 µg/mL as divisor. **C** ^1^DD of 4 and 12 µg/mL ASP in methanol (black) and plasma (red) using ROX 20 µg/mL and ^1^DD of 2 and 4 µg/mL ROX in methanol (black) and plasma (red) using ASP 36 µg/mL as divisor.

Standard addition technique was applied for the study by spiking suitable increments of each drug to blank plasma. This technique is more advisable for drugs with low plasma concentration levels since it augment the drugs’ concentrations to reach their Cmax [47, 48] and facilitate their quantitative analysis in human fluids. The matrix effect was assessed by comparing the absorption spectrum of blank plasma and the obtained absorption spectra of the prepared mixtures of ASP & ROX either in plasma or in methanol in the spiked concentrations (zero order absorption spectra, the ratio spectra after division by ROX 20 µg/mL spectrum or 36 µg/mL ASP spectrum and the first derivative ratio spectra), Fig. 6A–C. All these spectra proved the applicability the proposed methods for the determination of the selected drugs in the biological matrix of plasma without any interference at the selected wavelengths for measurements. Furthermore, Table 2 evinces the mean recoveries and RSD% of both drugs that support methods credibility.

### Statistical analysis

The obtained results of the suggested methods for determination of ASP and ROX were sufficiently good. Nonetheless, statistical evaluation of these results remains crucial, as it offers insight into whether the methods can be applied in QC & bioequivalent analysis. Statistical comparison with results obtained by applying the USP official method for ASP [40] and the reported HPLC method [34] was performed as shown in Table 3. Regarding t- and F-values, the null hypothesis was accepted, and the proposed methods showed good accuracy and excellent precision with no significant interference from pharmaceutical or biological matrices.

**Table 3:** Statistical analysis of the results obtained by the developed spectrophotometric methods and official and reported methods for determination of ASP and ROX in their pure powdered form

Parameter	ASP				ROX			
	DW	RD	1DD	Official^a^	DW	RD	1DD	Reported ^b^
Mean	100.26	100.11	99.7	100.22	100.06	100.24	99.67	100.48
SD	1.00	1.05	0.89	0.95	0.97	0.68	0.706	0.92
n	10	10	10	6	8	8	8	6
Variance	1.00	1.11	0.79	0.90	0.94	0.47	0.49	0.84
Student’s t-test	0.078 (2.145)*	0.205 (2.145)*	1.215 (2.145)*		0.855 (2.179)*	0.562 (2.179)*	1.888 (2.179)*	
F	1.10 (4.77)*	1.23 (4.77)*	1.14 (4.77)*		1.11 (4.88)*	1.79 (4.88)*	3.81 (4.88)*	

^*^The figures in parenthesis are the corresponding theoretical values at P = 0.05

^a^Official titrimetric USP method

^b^Reported HPLC method using C18 column isocratic elution using ACN: H_2_O (55:45 V/V), UV detection at 249 nm

## Methods comparative study

The applied spectrophotometric methods offer competitor analytical tools for the determination of such a new mixture. Despite some limitations for each method, they are considered the superior choice in QC labs, bioavailability labs, Table 4.

**Table 4:** Advantages and outcomes of the proposed spectrophotometric methods

Method	Advantages & outcomes	Limitations
DW	• Can determine the co-administered ASP &ROX successfully in different matrices • Applied on zero order spectra, consequently less noise is obtained • No need for further manipulation so, it does not require special software • Has the least mathematical slope & intercept of linear equation, so low interference and error index are assured • Has RSD% less than 2 which offers accurate and precise determinations	• Applied on Zero order spectra, so some minor features of the spectra may be neglected • The wavelength pairs of choice may be critical which propose meticulous analysis
RD	• Can determine the co-administered ASP &ROX successfully in different matrices • Applied on ratio spectra, that keeps the potential interference constant throughout the whole spectrum • Allow wide choice of wavelength pairs for determination • Critical determinations are avoided • Has low mathematical slope & intercept of linear equation, so low interference and error index are obtained • Has RSD% less than 2 which offers accurate and precise determinations	• Ratio spectra may enhance base line noise • It needs one more manipulation step • It requires divisor selection optimization
1DD	• Can determine the co-administered ASP &ROX successfully in different matrices • Applied on ratio spectra, that keeps the potential interference constant throughout the whole spectrum • Derivatization cancels the interfering contribution throughout the spectra • Derivative manipulation may enhance some minor features that permit clear determination • Allow variable wavelengths for quantitation • Has low mathematical slope & intercept of linear equation, so low interference and error index are obtained • Creates new sensitive analytical signals giving the chance of more sensitive results • Has RSD% less than 2 which offers accurate and precise determinations	• Ratio spectra with derivative steps may enhance base line noise • It needs more manipulation steps • It requires divisor and derivatization parameters optimization

## Ecological consideration and greenness assessment

**Fig. 7:**
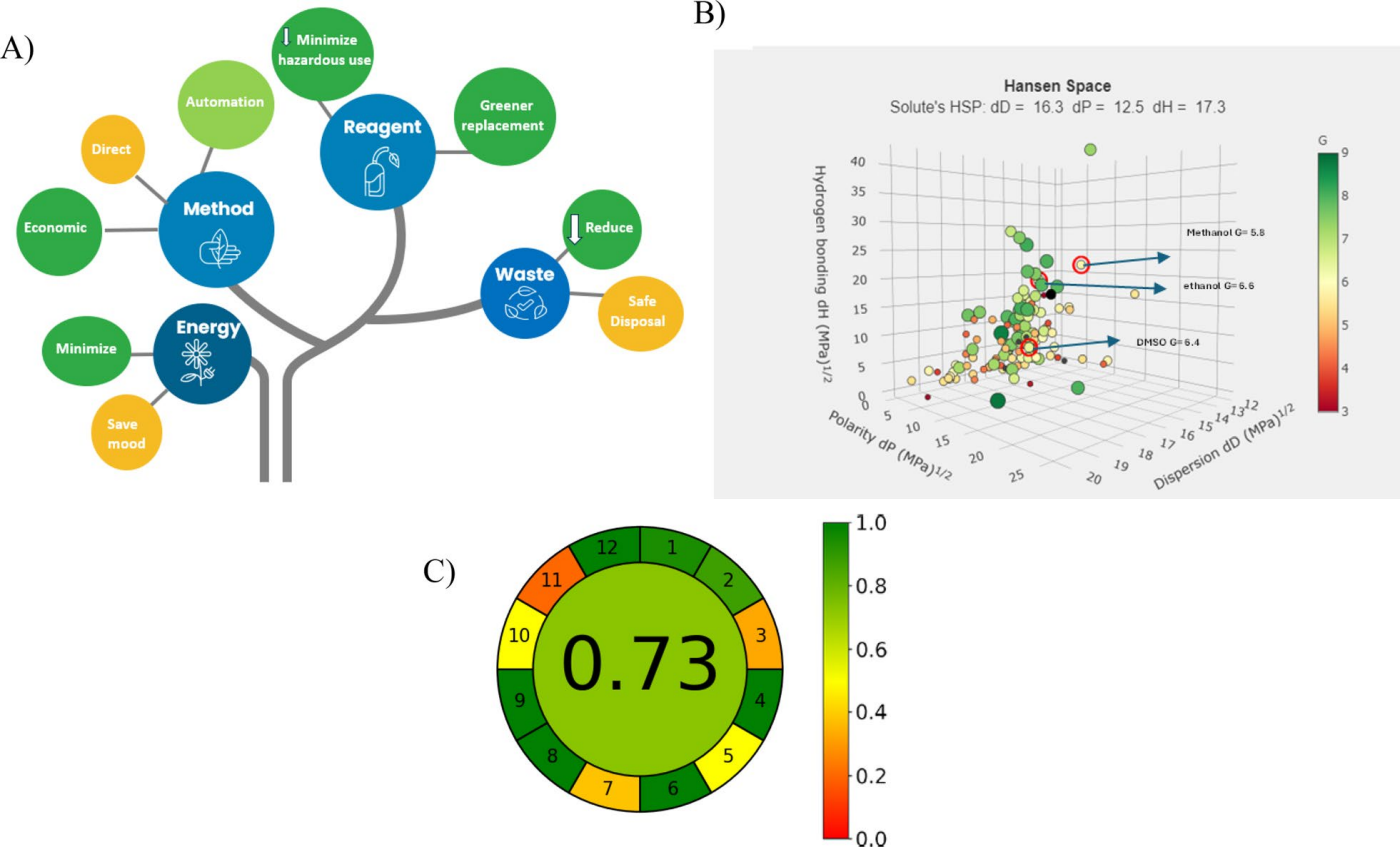
**A** Contemplating selection factors for the suggested method within the ecological paradigm framework. **B** G scores by GSST for ethanol, methanol and DMSO. **C** The Analytical Greenness Calculator (AGREE) for Greenness assessment

During the planning stage of the analytical methodology, great attention was given to evaluate its ecological impact [49]. Figure 7A elaborates the selection factors considered for the suggested method within the ecological paradigm framework. We chose a direct spectrophotometric method, as it offered a significant advantage of eliminating cumbersome sample preparation and separation steps, resulting in reduced use of hazardous solvents compared to Liquid chromatographic methods [15, 50].

For the solvent selection, it was the most crucial step. Rivaroxaban solubility is challenging as it is water insoluble and sparingly soluble in alcohols like ethanol and methanol. This was overcome by use of co-solvents to improve solubility without excessive use of the less green solvents. After several solubility trials, we have selected to use methanol with minimum volume of DMSO that guarantee a full solubility of the drug even after storage proved by intermediate precisions and stability checking of the stock solution. This practice echoed the recommendation of rivaroxaban solubility reported in [51] that recommends the binary solvents mixture of DMSO and Methanol. We used the greenness score from the GSK Solvent Sustainability Guidelines [52], which presents a numerical evaluation of solvents through a Composite Score reflecting various criteria (G) which calculated using a free calculator http://green-solvent-tool.herokuapp.com/ according to the equation G = ∜(H × S × E × W). Ethanol scored G = 6.6 while DMSO 6.4 and Methanol 5.8 (Fig. 7B). However, the use of ethanol, a more environmentally friendly option but was not conducive to the drug’s solubility. This necessitates a balance between adhering to eco-friendly practices and ensuring the method's practicality. In addition, the use of methanol facilitates the plasma extraction procedure [53] as it acts as the precipitating agent for the proteins without the need of various reagents with various environmental impacts. Any interference from the used solvent can simply be removed using a blank.

In terms of energy use, being spectrophotometric methods, we consumed minimal energy due to quick sample measurements and the offline spectra analysis and manipulation. Moreover, for the produced waste, compared to alternative chromatographic techniques, it generated significantly less waste, making safe waste disposal easier to safeguard.

To assess the greenness profile, we have used the latest developed tool, the Analytical Greenness calculator (AGREE) [54]. It utilizes a simple algorithm that uses a scale from 0 to1 to represent the method’s overall environmental sustainability, along with a pictorial representation directly linked to the 12 GAC rules. The automatically generated pictogram is composed of twelve sections, each featuring a unique color gradient that spans from dark green (value = 1) to dark red (value = 0). At the center of the pictogram, the total score is displayed as a fraction, with values ranging from zero to one indicating how close to the ideal green value (value = 1). The AGREE software delivered a detailed assessment of the entire analytical process associated with each principle of green chemistry and the calculation is automated using free software (https://mostwiedzy.pl/AGREE), [54]. It emphasized the most vulnerable segments of the analytical procedures, pinpointing where further adjustments are necessary to enhance sustainability. Our suggested spectrophotometric methods proved its agreement with the GAC principles through a comprehensive assessment of its efficiency, practicality, and environment friendliness. The Agree score is 0.73, the missing points are mainly related to miniaturization and the reagents which cannot be fully avoided especially with the solubility issues of the studied drug. The score still ensures an overall minimal ecological footprint of the suggested methods as shown in Fig. 7C.

## Concluding remarks

The recommended methods are considered the first spectrophotometric methods to determine this novel combination therapy of aspirin and rivaroxaban. They are selective, accurate, sensitive, and more appropriate, easier, and cheaper for determination of the studied mixture when compared to any chromatographic method. Even though the Cmax does not lie within the working concentration range of the suggested methods, applying the spiking technique allows their accurate analysis. The proposed methods provide a direct, cost effective and simple alternative for determination of the combination therapy without preliminary separation or elaborate treatment associated with chromatographic methods.

Linearity of the calibration graphs and adherence to Beer’s law were confirmed by the small intercepts and the high values of correlation coefficients. The three methods demonstrated reliability and accuracy, with minimum data manipulation requirement. The DW method was the simplest, while the DR method has broader application as it does not require constant absorbance of the interfering components at the selected wavelengths. The suggested methods were successfully applied to the analysis of ASP and ROX in spiked human plasma.

## Data Availability

Any supplementary data will be made available on request.
